# Diacetyl Vapor Inhalation Induces Mixed, Granulocytic Lung Inflammation with Increased CD4^+^CD25^+^ T Cells in the Rat

**DOI:** 10.3390/toxics9120359

**Published:** 2021-12-20

**Authors:** Emma L. House, So-Young Kim, Carl J. Johnston, Angela M. Groves, Eric Hernady, Ravi S. Misra, Matthew D. McGraw

**Affiliations:** 1 Department of Pathology, University of Rochester Medical Center, Rochester, NY 14642, USA; emma_house@urmc.rochester.edu; 2 Division of Pediatric Pulmonology, Department of Pediatrics, School of Medicine & Dentistry, University of Rochester Medical Center, Rochester, NY 14642, USA; soyoung_kim@urmc.rochester.edu (S.-Y.K.); angela_groves@urmc.rochester.edu (A.M.G.); 3 Department of Environmental Medicine, School of Medicine & Dentistry, University of Rochester Medical Center, Rochester, NY 14642, USA; carl_johnston@urmc.rochester.edu (C.J.J.); eric_hernady@urmc.rochester.edu (E.H.); 4 Department of Radiation Oncology, University of Rochester Medical Center, Rochester, NY 14642, USA; 5 Division of Neonatology, Department of Pediatrics, University of Rochester Medical Center, Rochester, NY 14642, USA; ravi_misra@urmc.rochester.edu

**Keywords:** CD4^+^CD25^+^ T cells, lung, airways, diacetyl, 2,3-butanedione, flavorings-related lung disease, hazard potential, intoxication, occupational hazard, bronchiolitis obliterans

## Abstract

Diacetyl (DA) is a highly reactive alpha diketone associated with flavoring-related lung disease. In rodents, acute DA vapor exposure can initiate an airway-centric, inflammatory response. However, this immune response has yet to be fully characterized in the context of flavoring-related lung disease progression. The following studies were designed to characterize the different T cell populations within the lung following repetitive DA vapor exposures. Sprague-Dawley rats were exposed to 200 parts-per-million DA vapor for 5 consecutive days × 6 h/day. Lung tissue and bronchoalveolar lavage fluid (BALF) were analyzed for changes in histology by H&E and Trichrome stain, T cell markers by flow cytometry, total BALF cell counts and differentials, BALF IL17a and total protein immediately, 1 and 2 weeks post-exposure. Lung histology and BALF cell composition demonstrated mixed, granulocytic lung inflammation with bronchial lymphoid aggregates at all time points in DA-exposed lungs compared to air controls. While no significant change was seen in percent lung CD3^+^, CD4^+^, or CD8^+^ T cells, a significant increase in lung CD4^+^CD25^+^ T cells developed at 1 week that persisted at 2 weeks post-exposure. Further characterization of this CD4^+^CD25^+^ T cell population identified Foxp3^+^ T cells at 1 week that failed to persist at 2 weeks. Conversely, BALF IL-17a increased significantly at 2 weeks in DA-exposed rats compared to air controls. Lung CD4^+^CD25^+^ T cells and BALF IL17a correlated directly with BALF total protein and inversely with rat oxygen saturations. Repetitive DA vapor exposure at occupationally relevant concentrations induced mixed, granulocytic lung inflammation with increased CD4^+^CD25^+^ T cells in the rat lung.

## 1. Introduction

Diacetyl, (2,3-butanedione; DA) is a highly reactive alpha diketone used in commercial food manufacturing as an additive for its buttery flavor and aroma. DA also occurs naturally through coffee roasting and alcohol fermentation [1,2,3]. Due to its low boiling point (88 °C), DA frequently enters the vapor phase during the production process. DA is highly reactive due to its inherent chemical properties. It has two strong electrophilic carbonyl moieties that can easily and non-enzymatically modify nearby nucleophilic residues in adjacent cellular proteins [4]. These two chemical properties make DA a significant respiratory hazard. Over the past two decades, DA vapor exposures has been well associated with the fibrotic lung disease known as bronchiolitis obliterans (BO), now labeled as “flavoring-related lung disease” [5]. BO is defined by histopathology with significant small airways, or bronchiolar, remodeling consisting of sub-epithelial collagen deposition and subsequent airway lumen occlusion due to circumferential narrowing [6]. Despite multiple reports of severe lung disease following DA exposure, DA remains a significant and relevant respiratory toxicity considering its more recent identification in other foods such as coffee roasting and e-cigarettes aerosols [1,7,8].

Preclinical studies of flavoring-related lung disease reveal primary injury to the airway epithelium as the sentinel event to disease pathogenesis [9,10,11]. Severe injury of the airway results in ulceration and necrosis of the epithelium, injury to the adjacent basement membrane, and subsequent initiation of an inflammatory and fibro-proliferative response. With sufficient injury, abnormal repair of the airway epithelium often occurs with epithelial hyperplasia and concentric overgrowth of sub-epithelial fibrous tissue [9,10]. This injury and abnormal epithelial repair initiates a potent inflammatory response seen in both mice and rats exposed repetitively to DA vapors [11,12]. Neutrophils are most frequently observed within the bronchoalveolar lavage fluid (BALF) as well as adjacent lamina propria of the airways. Other infiltrative cell types include lymphocytes and rare eosinophils [12,13]. BALF neutrophilia can persist for weeks after DA exposure despite exposure cessation [9,14]. This persistent BALF neutrophilia is consistent with those workers exposed to high concentrations of DA at popcorn factories as well as other individuals identified to have BO pathology [5]. Thus, this mixed granulocytic inflammation with BALF neutrophilia is an important characteristic of flavoring-related lung disease.

One associated but less characterized attribute of flavoring-related lung disease pathology is the adaptive immune response. Increased peribronchial lymphocytic aggregates have been noted in both mice and rats exposed to DA [11,12]. Additionally, mice exposed to 2,3-pentanedione, a chemically similar and highly reactive ketone, showed frequent nodular and linear lymphoid aggregates adjacent to exposed bronchioles and bronchi, supportive of a localized T cell response [15]. Most of the lymphoid growth seen after inhalation exposure is adjacent to injured airways with less growth of peripheral lymphoid aggregates. In one rare abstract, the T cell response was evaluated in athymic nude rats exposed to DA intratracheally [16]. Thymus-deficient rats exposed to DA developed worse airways pathology compared to DA-exposed rats alone [16], indicating a potentially protective effect of the adaptive immune response in DA-exposed animals with previous thymic resection.

The primary goal of the following studies was to further characterize the T cell phenotypes within the lung and their association with flavoring-induced lung disease progression. For studying the immune response, our lab previously developed an in vivo vapor exposure system using DA with a 14-day follow-up period for modeling the adaptive immune response in flavoring-induced lung disease [14]. We used this model for analyzing the proportions of CD3^+^, CD4^+^, CD8^+^, CD4^+^CD25^+^ and CD4^+^CD25^+^Foxp3^+^ (regulatory T cells; T_regs_) immediately following DA exposure (Day 5), 1 week (Day 12) and 2 weeks (Day 19) post-exposure. We also analyzed bronchoalveolar fluid at these specified time points for changes in expression of the CD4^+^CD25^+^ T cell-related cytokine IL17a. We hypothesized that the balance of different CD4^+^CD25^+^ T cell populations within the lung contribute to the progression of flavoring-related lung disease.

## 2. Materials and Methods

### 2.1. Animals

All studies were approved by the Institutional Animal Care and Use Committee of the University of Rochester Medical Center (URMC). Investigators adhered to the National Institute of Health (NIH) Animal Welfare guidelines. Male outbred six to eight-week-old Sprague–Dawley rats (Charles River Laboratory, Wilmington, WA, USA) weighing between 200–250 g were maintained in an AALAC-accredited facility.

### 2.2. Diacetyl Vapor Exposures

Whole body exposures to diacetyl (DA, 2,3-butanedione; >98% pure; Sigma-Aldrich, St. Louis, MO, USA) were performed at the URMC inhalation exposure Facility (IEF), as described in the previous publication [14]. Briefly, animals were exposed to 200 parts-per-million (ppm) DA vapor in a whole-body exposure chamber for 5 consecutive days, 6 h/day. Weight and oxygen saturation (Starr Life Science Technologies, Oakmont, PA, USA) were monitored throughout the duration of the exposure and during a 14-day period following exposure. Animals were euthanized at Day 5 (immediately after last DA exposure), Day 12 (1 week post-exposure), and Day 19 (2 weeks post-exposure) for tissue harvest. Three (3×) separate DA exposures were performed for exposure replicates with representative datasets included for publication.

### 2.3. Bronchoalveolar Lavage Fluid (BALF), Tissue Harvest, and Histopathology

Animals were euthanized with an intraperitoneal injection of Euthasol (pentobarbital sodium and phenytoin sodium, Virbac, France). The descending aorta was transected for as a form of secondary euthanasia. The right ventricle was then injected with 10 mL 0.9% normal saline solution for lung perfusion. The trachea was cannulated with an 18 G cannula. The left mainstem bronchus was tied off, and the right lungs were lavaged with 5.0 mL 0.9% normal saline solution (bronchoalveolar lavage fluid, BALF). BALF samples were centrifuged at 200× *g* for 10 min at 4 °C, and supernatants frozen (−80 °C) for future analyses of 1 mL aliquots.

The right lung lobes were isolated and suture tied in order for the left lung lobes to be fixed in 10% neutral buffered formalin and later transferred to 70% ethanol. Lung tissue was embedded in paraffin, sectioned (5 µm) and stained with hematoxylin and eosin (H&E) or Masson’s Trichrome. Lung tissue was also stained for the T cell marker CD3 (1:200, C7930, MilliporeSigma, St. Louis, MO, USA) or rabbit IgG (1:200, Agilent, Santa Clara, CA, USA; negative control). Staining in rat spleen was used as a CD3 positive control. In brief, sections were deparaffinized and rehydrated through ethanol. Heat-mediated antigen retrieval was performed in citrated retrieval solution for 30 min (Agilent). Slides were blocked for 1 hour with Rodent Block R (Biocare Medical, Pacheco, CA, USA) and then primary antibody Cambridge, UK) was applied for 1 h, and developed using DAB Chromagen (Biocare Medical, Pacheco, CA, USA). Slides were then counterstained with hematoxylin, dehydrated, and finished with cover slip.

Representative images of affected airways were taken from each left lung at sequential post-exposure time points. Lung scoring for airway lymphoid hyperplasia and sub-epithelial matrix deposition was performed on embedded lung tissue to semi-quantitate the changes in airway architecture observed on light microscopy [17]. Briefly, intrapulmonary bronchi were identified within each rat lung section, defined by a luminal diameter greater than 250 µm and not connecting directly to the alveolar space [10,15]. Five bronchi were graded for each rat at 4× (bar: 250 µm) (5 rats/time point × 5 bronchi/rat). For airway remodeling, the presence or absence of increased sub-epithelial, extracellular matrix was noted by Masson’s Trichrome. For lymphoid hyperplasia, each aggregate adjacent to the bronchi was graded with: 0 for absence of an aggregate, 1+ for 1/3, 2+ for <2/3, and 3+ for >2/3 of the field obstructed by the aggregate. The cumulative average and standard deviation was then calculated for each time point.

### 2.4. Flow Cytometry for T Cells in Whole Lung Homogenates

The right medial lung was excised and placed in digestion buffer of 5% FBS and 7.5 µg/mL collagenase A (Roche, Basel, Switzerland) in PBS on ice for 30 min. Tissue was further dissociated using a GentleMacs (Miltenyi Biotec, Auburn, CA, USA) Tissue dissociator followed by needle aspiration through an 18 G needle and syringe. Afterwards, three aspiration homogenates were expelled onto 70 µM filter and disrupted with syringe plunger. To ensure maximal cell retrieval filters were washed with ice cold FACS buffer containing 5% FBS in PBS. After spinning for 5 min at 300× *g*, supernatants were discarded and pellets re-suspended ammonium-chloride-potassium (ACK) buffer (Thermo Scientific, Waltham, MA, USA; Cat # A1049201) for red blood cell lysis prior to a final wash. Cell suspensions were adjusted to a concentration of approximately 1 × 10^7^ cells/mL in FACS buffer for flow cytometric staining.

Single cell suspensions of whole lung homogenates were first stained for surface markers: CD3, CD8, CD4, and CD25 (see Supplemental Table S1). Next intracellular staining for Foxp3 was completed using Foxp3 fixation/permeabilization kit (eBioscience, San Diego, CA, USA; Cat. #2506527), per manufacturer’s instructions. Complete information about clone and dilutions of antibodies used are also available in Table S1. LSR II flow cytometer (BD Biosciences, Franklin Lakes, NJ, USA) was used at the URMC Flow Cytometry Core (URMC FCR) to acquire data. FCS express (De Novo Software, Pasadena, CA, USA, version 7) was used to analyze data captured. Gates were set based on side scatter area (SSC-A) versus forward scatter area (FSC-A) to exclude debris and then sub-gated for forward and side scatter height to isolate single cells. CD3^+^ T cells were then isolated from this single cell population. Sub-gates were applied to resolve the CD4^+^ and CD8^+^ T cell populations from this parent gate. Downstream CD4^+^CD25^+^ and CD4^+^CD25^+^FoxP3^+^ T cell populations were also defined from this parent population.

### 2.5. Bronchoalveolar Fluid (BALF) Total Protein and Interleukin-17A (IL-17a)

Bronchoalveolar lavage fluid (BALF) was collected from all animals for total protein quantification. Total protein was determined using the Pierce bicinchoninic acid (BCA protein) assay kit (Thermo Scientific; Cat # PI23225). BALF IL-17a was determined using a Duoset ELISA immunoassay (R&D Systems, Minneapolis, MN, USA; Cat # DY8410-05). Standards and assays were prepared and performed according to manufacturer’s instructions. Briefly, BALF was centrifuged at (300× *g*, 5 min) to remove debris. Supernatant was decanted and aliquots were applied to prepare for quantification.

### 2.6. Statistical Analysis

Linear regression modeling was performed (SAS v9.4, Cary, NC, USA) to estimate the sample size with the primary end-point being percent change in CD4CD25^+^ T cells at Day 12 (‘inflammatory period’) when accounting for differences in survival. We assumed the average percent CD4CD25^+^ T cells in the lung to be 8% with a standard deviation of 3% and a 50% increase (4% total change) with DA exposure [18]. With an alpha of 0.05 and power of 80%, the number of animals per group was 9 (total number: 36). Thus, we performed 3 exposure replicates (5 animals/group), accounting for animal loss. Prism v8.0 (GraphPad Software, San Diego, CA, USA) was used for post-exposure statistical analysis with ANOVA followed by correction for multiple comparisons assuming normal sample distribution while Kruskal–Wallis tests with Dunn’s correction was performed for skewed samples.

## 3. Results

### 3.1. Mixed, Granulocytic Airways Inflammation with Adjacent Lymphoid Aggregates Following DA Vapor Exposures

**Figure 1:**
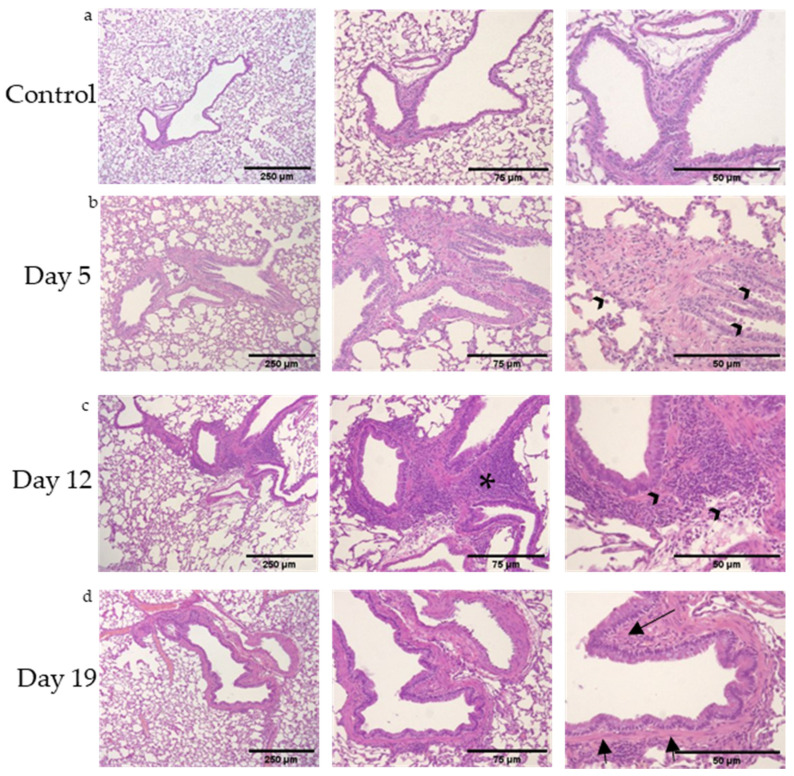
Representative rat lung tissue stained for hematoxylin and eosin (H&E) (bar: 250 µm (left column), 75 µm (middle column), and 50 µm (right column)). (**a**) Room air (control)-exposed rat airways, (**b**) Day 5 (immediately post-exposure) rat airways, (**c**) Day 12 (1 week following DA exposures) rat airways, and (**d**) Day 19 (2 weeks post-exposure) rat airways. Lymphoid aggregates are seen adjacent to affected airways with perivascular and bronchiolar edema (asterisk). Bronchial walls thickening identified at Day 12 and 19 (arrowheads and arrows, respectively).

Rat lungs were evaluated by histology for airways inflammation immediately after exposure (Day 5), 1 week post-exposure (Day 12) and 2 weeks post-exposure (Day 19); Figure 1. In all DA-exposed rats, intrapulmonary bronchi appeared with circumferential airways inflammation as early as Day 5 post-DA exposure that persisted until Day 19 in DA-exposed airways (n = 20 rats/group with >5 airways assessed/animal). Circumferential airways inflammation was most prominent 1 week post-exposure (Day 12, Figure 1c, asterisks). By Day 19, thickening of the bronchial walls with deposition of sub-epithelial, extracellular matrix was seen on H&E (Day 19, Figure 1d, arrows) and Masson’s Trichrome (Figure S1). The number of bronchi affected by thickened sub-epithelial, extracellular matrix and lymphoid hyperplasia was semi-quantitated at each time point after DA exposure (n = 5 rats × 5 bronchi/time point, Table 1). Both histologic parameters increased with respect to time after DA exposure.

In line with previous in vivo DA exposures [9,10], airways inflammation consisted of mixed, granulocytic lung inflammation with a predominance of neutrophils, lymphocytes, macrophages and rare eosinophils (higher magnification image, Figure S2). Immunohistochemical staining for CD3, a common T cell marker, also identified that these airway-centric, lymphoid aggregates stained positive (Figure S3). Collectively, these histopathologic findings support DA inducing mixed, granulocytic lung inflammation with airway remodeling and lymphoid hyperplasia adjacent to affected airways.

### 3.2. Bronchoalveolar Lavage Fluid (BALF) Cell Counts and Differentials after DA Exposures

**Figure 2:**
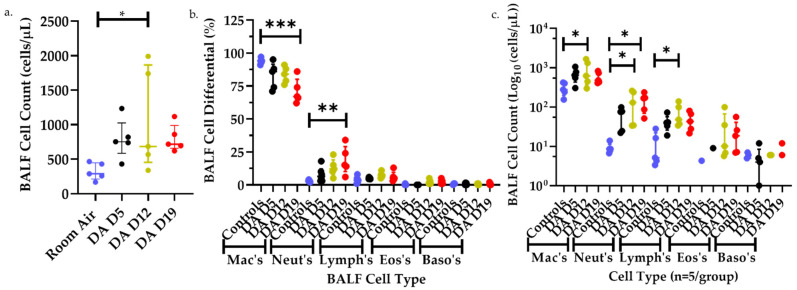
BALF total cell counts (**a**), percent cell differentials (**b**), and individual cell counts (**c**) from room air (blue), DA-exposed Day 5 (black), Day 12 (yellow), and Day 19 (red). (**a**) BALF total cell counts increased significantly in DA D12 compared to room air controls (Kruskal–Wallis, * *p* < 0.05). (**b**) BALF percent macrophages decreased significantly in DA D19 compared to room air controls (ANOVA, *** *p* < 0.001) while percent BALF neutrophils increased significantly in DA D19 rats compared to controls (ANOVA, ** *p* < 0.01). (**c**) Total BALF macrophages, neutrophils and lymphocytes increased significantly in DA D12 rats compared to controls (ANOVA, * *p* < 0.05), and BALF neutrophils increased significantly in DA D19 compared to controls (ANOVA, * *p* < 0.05).

To provide additional support of the inflammatory changes seen by histology, the total number of cells, percent cell differentials, and individual cell numbers by cell type were performed on BALF from room air, DA D5, D12, and D19 rat lungs (Figure 2). The total number of BALF cells increased significantly in DA-exposed animals 1 week after DA exposure (DA D12) compared to room air controls but did not differ significantly at DA D5 or DA D19 (Figure 2a). The percentage of BALF macrophages decreased significantly in DA-exposed animals at D19 compared to controls while the percent BALF neutrophils increased significantly in DA-exposed animals at D19 compared to air controls (Figure 2b). The percent BALF lymphocytes, eosinophils and basophils did not differ significantly between groups, however, the total number of BALF macrophages, neutrophils and lymphocytes increased significantly at D12 in DA-exposed animals compared to controls (Figure 2c). Hence, changes in BALF cell number and composition further support a mixed, granulocytic infiltrate within the rat lung after DA exposure composed predominantly of neutrophils and lymphocytes and peaking 1 week after DA exposure cessation (DA D12).

### 3.3. T Cell Populations within the Rat Lung after DA Exposure

**Figure 3:**
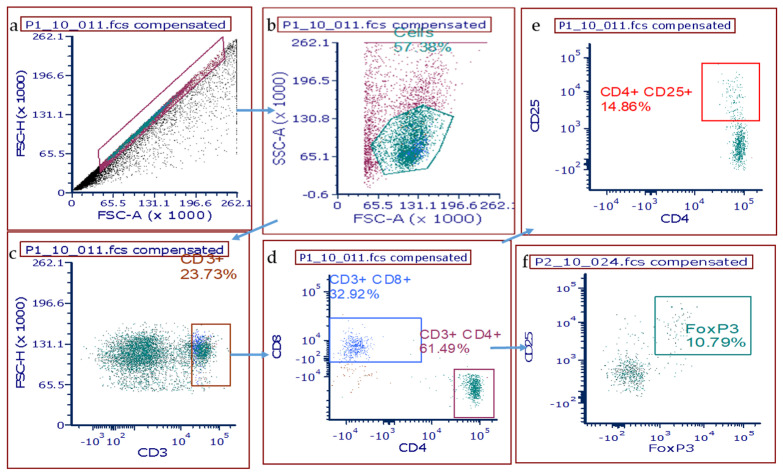
Gating scheme for T cell isolation and identification from whole rat lung homogenates. (**a**) Total isolated cells were first gated for singlets on FSC−A versus (vs.) FSC−H, then (**b**) SSC−A vs. FSC−A to exclude debris and followed by (**c**) CD3^+^ cells vs. FSC−H. (**d**) Sub-gating for CD8+ or CD4+ sub-populations was then applied, and then further gated for CD25+ (**e**) and FoxP3+ T cell populations (**f**).

In light of the lymphoid hyperplasia seen on histology, flow cytometry was performed on whole lung homogenates to evaluate changes in T cell populations after DA exposures. Lung homogenates were stained for the common T cell surface markers CD3, CD4, CD8 and CD25 as well as the intracellular stain Foxp3. A schematic representation of flow gating is presented in Figure 3. Briefly, cells were gated first for size and granularity (SSC-A/FSC-A; FSC-H/FSC-A) to remove debris and “doublets”, respectively. The CD3^+^ population was then sub-gated directly from the singlet population. Next, CD4^+^ and CD8^+^ cell populations were discriminated by sub-gating of the CD3^+^ parent population. Finally, gates were applied to discriminate the CD4^+^CD25^+^ and CD4^+^CD25^+^Foxp3^+^ populations.

**Figure 4:**
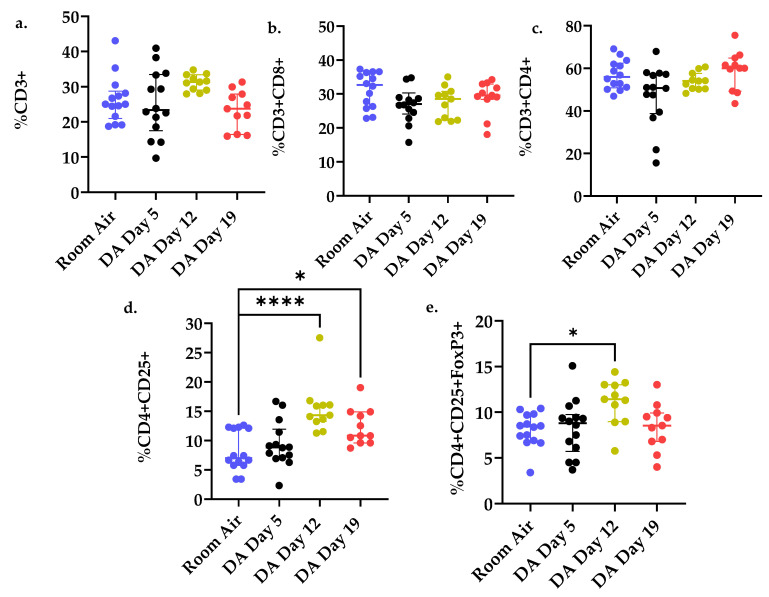
Percent (%) T cell populations from rat lung homogenates exposed to room air (blue) versus DA-exposed Day 5 (black), Day 12 (yellow), and Day 19 (red). (**a**) CD3^+^%, (**b**) CD3^+^CD8+%, (**c**) CD3+CD4+%, (**d**) CD4+CD25+%, and (**e**) CD4^+^CD25^+^Foxp3^+^% lung T cells. * *p* < 0.05, ** *p* < 0.01, **** *p* < 0.0001. Cumulative results from 3 separate exposures (n = 11–14/group); differences in group size are due to animal death at later time points (D12, D19).

No significant difference in percent lung CD3^+^, CD4^+^, and CD8^+^ T cell populations was seen between DA- and air-exposed rats at any of the time points assessed after DA exposure (one-way ANOVA; *p* > 0.05, Figure 4a–c respectively). However, the percent of CD4^+^CD25^+^ T cells increased significantly at Day 12 and remained increased at Day 19 in DA-exposed compared to air controls (ANOVA with Dunnett’s, **** *p* < 0.0001; * *p* < 0.05, Figure 4d). Conversely, staining for Foxp3^+^ T cell expression revealed a significant increase at Day 12 in DA-exposed compared to air-exposed controls that did not persist until Day 19 (ANOVA with Dunnett’s, ** *p* < 0.01, Figure 4e).

### 3.4. Increased IL-17a Expression and Total Albumin in Bronchoalveolar Lavage Fluid (BALF) after DA Exposure

**Figure 5:**
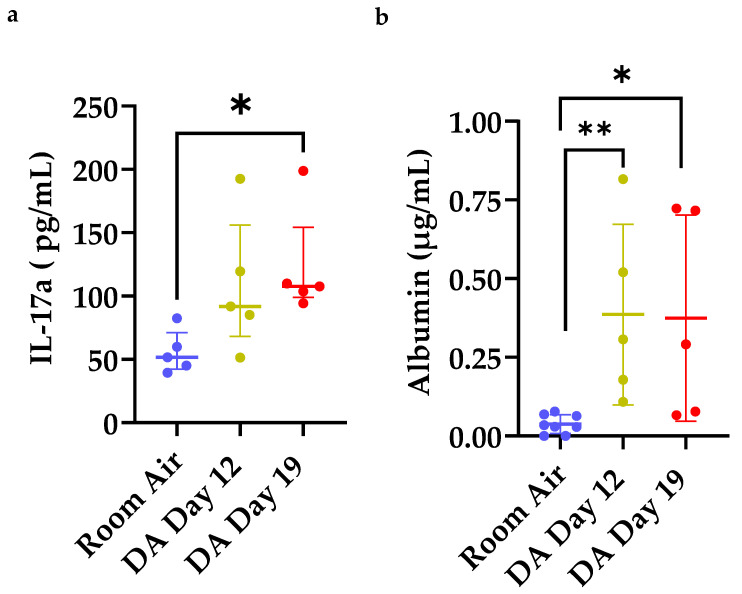
Bronchoalveolar lavage fluid (BALF) expression of (**a**) IL-17a and (**b**) albumin in room air (blue) and DA-exposed rats at Day 12 (yellow) and Day 19 (red). (**a**) BALF IL-17a expression increased significantly in DA D19 rats compared to room air controls (Kruskal–Wallis, * *p* < 0.05). (**b**) BALF albumin increased significantly in both DA D12 and D19 compared to room air controls (Kruskal-Wallis, ** *p* < 0.01 and * *p* < 0.05).

Next, we evaluated for the presence of IL-17a protein expression and total albumin in BALF fluid at Day 12 and 19 after DA exposure (Figure 5). IL-17a is a pro inflammatory cytokine released by effector CD4^+^CD25^+^ cells under certain inflammatory diseases and associated with other forms of bronchiolitis obliterans [19,20]. A significant increase in BALF IL-17a expression was seen at Day 19 in DA-exposed rats compared to room air controls (* *p* < 0.05, Figure 5a). BALF albumin, a surrogate of lung permeability, also increased at Days 12 and 19 in DA-exposed animals compared to room air controls (* *p* < 0.05, Figure 5b). The increase in BALF IL-17a expression coincided temporally with the persistence of lung CD4^+^CD25^+^ T cells (Figure 4d), decrease in lung CD4^+^CD25^+^Foxp3^+^ T cells (Figure 4e) and increase in BALF albumin after DA exposure (Figure 5b).

### 3.5. Percent CD4^+^CD25^+^ Lung T Cells Correlates with Reduced Oxygen Saturations and Increased Lung Permeability

**Figure 6:**
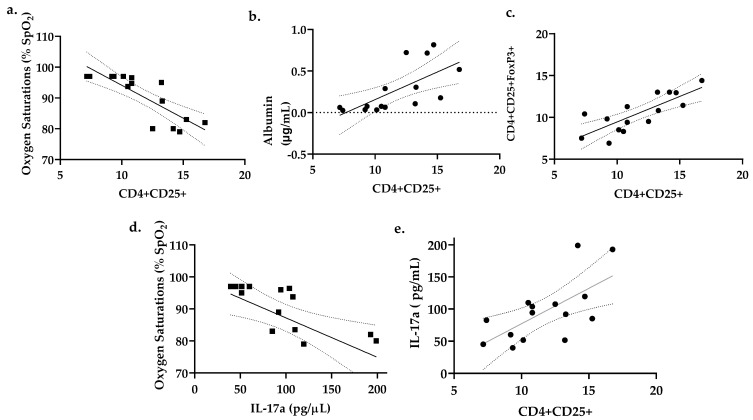
Pearson correlations between oxygen saturations, bronchoalveolar lavage fluid (BALF) albumin, BALF IL17a expression, and percent (%) total lung CD4^+^CD25^+^ T cells after diacetyl inhalation exposures. (**a**) Oxygen saturations correlated inversely with % lung CD4^+^CD25^+^ T cells (*r* = −0.7320, *p* < 0.001). (**b**) BALF albumin correlated directly with percent lung CD4^+^CD25^+^ T cells. (*r* = 0.8942, *p* < 0.01). (**c**) Foxp3 positive T cells correlated directly with CD4^+^CD25^+^ T cells. (*r* = 0.796, *p* = 0.0005) (**d**) Day 19 oxygen saturations correlated inversely with BALF IL-17a concentration. (*r* = 0.5315; *p* < 0.01). (**e**) BALF IL-17a correlated directly with percent lung CD4^+^CD25^+^ T cells (*r* = 0.6828; *p* = 0.0001).

To further characterize the physiologic consequences of increased CD4^+^CD25^+^ T cells within the lung after DA exposure, correlations were performed comparing percent lung CD4^+^CD25^+^ T cells, BALF albumin, BALF IL17a expression and percent oxygen saturations (% SpO_2_) in DA-exposed animals (Figure 6). In our model of repetitive DA vapor exposures, oxygen saturations fell precipitously in DA-exposed rats at DA Day 12 and remained abnormal (below 90%) until study’s end (Day 19) [14]. An inverse correlation was seen between percent lung CD4^+^CD25^+^ T cells and oxygen saturations at Day 12 (Figure 6a; *r* = −0.6755; *p* < 0.001). A positive correlation was observed at both Days 12 and 19 between percent lung CD4^+^CD25^+^ T cells to BALF albumin (Figure 6b; *r* = 0.4490; *p* < 0.01). While a direct correlation was seen between the percent CD4^+^CD25^+^ and Foxp3^+^ T cells (Figure 6c), no significant correlation was found between the percent lung CD4^+^CD25^+^Foxp3^+^ T cells and oxygen saturations. Conversely, an inverse correlation was seen between BALF IL-17a and oxygen saturations at D12 and D19 (Figure 6d; *r* = 0.5315, *p* < 0.01). Finally, a positive correlation between percent lung CD4^+^CD25^+^ T cells and BALF IL-17a was also seen (Figure 6e; *r* = 0.6828, *p* = 0.0001). Collectively, these correlations suggest that this CD4^+^CD25^+^ T cell population found within the rat lung following DA exposure may contribute to worsening oxygen saturations and increasing lung permeability following repetitive DA vapor exposures.

## 4. Discussion

In the current study, we characterize the lung’s T cell response in flavoring-related lung disease progression. While the overall percent of CD3, CD4, and CD8 T cells did not change, the percent of lung CD4^+^CD25^+^ T cells increased significantly after repetitive DA exposures with the development of persistent hypoxemia, increased lung permeability, and sub-epithelial fibrin deposition circumferential to affected airways. More specifically, two populations of CD4^+^CD25^+^ T cells increased within the lung after exposure: Foxp3^+^ and IL17a-secreting T cells. Increases in expression of these two CD4^+^CD25^+^ T cell populations were inverse of one another with Foxp3^+^ cells increasing 1 week post-exposure and BALF IL17a expression increasing at 2 weeks post-exposure.

A limited number of previous studies support our current findings that airway-centric, mixed granulocytic inflammation precedes bronchial fibrosis in DA inhalation exposures [11,12,14]. One such study demonstrated increased both B and T cell aggregates in mice exposed repetitively to DA vapors [11]. However, these DA-exposed mice did not develop the pathologic bronchial fibrosis seen in DA-exposed rats. One potential explanation for this difference in rodent response is the morphologic differences of rats and mice airways. In rats, keratin 5 positive airway epithelial cells extend into the intrapulmonary airways of rat that are not seen frequently in mouse intrapulmonary airways [21]. A second study using the structurally similar, volatile flavoring chemical 2,3–pentanedione found increased circumferential and airway-centric lymphocytes in DA-exposed Sprague–Dawley rats [15]. Immunohistochemistry of DA-exposed rat lungs revealed a predominance of CD3^+^ T cells within these lymphoid aggregates, consistent with the current study of further characterization of the different T cell subpopulations within the lung after DA exposures.

In comparison to flavoring-related lung disease, histopathology seen transplant-associated BO remains more commonly studied. In transplant-associated BO, disease pathogenesis is often described in three phases: (1) initial airway injury, (2) inflammatory induction, and subsequent (3) fibroproliferation [22]. Prior studies in transplant-associated BO strongly support dysregulation of the inflammatory phase that impairs epithelial repair and promotes BO development [20]. More recently, the appropriate balance of Th17 and regulatory (Th17/T_reg_) T cells has been identified as critical in damage resolution. Additionally, skewing of this Th17/T_reg_ axis toward a Th17-preferred phenotype has been reported in patients suffering from post-transplant BO [20,23]. Hence, targeting of this axis has been used to successfully modulate experimental BO in mice post-allograft lung transplant [24]. Furthermore, IL-17a blockade partially attenuates BO pathology in allograft lung transplant [24]. Similarly, Fan et al. reported partial attenuation of BO pathology after IL-17a neutralization in mice [25]. Similarly, Shi and colleagues reported adoptive transfer of Foxp3^+^ T cells in a rat orthotropic tracheal transplant model reduced central airway obliteration and sub-epithelial fibrin deposition compared to control animals not receiving adoptive transfer [26]. While this Th17/T_reg_ axis is now well characterized in transplant-associated BO, the beneficial effect of regulatory T cells and/or detrimental effects of Th17 cells has yet to be established in the development of flavoring-induced airways disease.

Imbalance of the Th17/T_reg_ axis has also been implicated in the development of ‘mustard lung disease’ [27], another fibroproliferative, airways-centric lung disease following toxic chemical inhalation. In subjects previously exposed to sulfur mustard (SM), circulation levels of Foxp3 mRNA were lower than those levels found in healthy controls whereas mRNA levels of the Th17 transcription factor ROR-yt were increased in comparison to healthy control levels [28]. Additionally, Foxp3 mRNA levels correlated directly with the percent forced expiratory volume in 1 s (% FEV1), a common surrogate marker of lung function, while the inverse correlation was true for ROR-yt mRNA levels and %FEV1 in mustard lung subjects. Other Th17-related cytokines, including IL-17a, IL-1β, IL-23, and IL-6, were also elevated in the blood of sulfur mustard-exposed subjects with an inverse correlation with %FEV1 [27,28] Hence, dysregulation of the Th17/T_reg_ axis is a potential pathway common to both flavoring-related lung disease and mustard lung disease.

Imbalance of Th17/T_reg_ axis is similarly implicated in the immunopathogenesis of other inhalation-induced lung diseases, such as smoke-inhalation lung injury [29,30]. One such study found rats exposed to gun powder smoke, composed primarily of particulate matter 2.5 (PM2.5), experienced increased number of circulating and lung-specific Th17 cells with decreased Foxp3^+^ T cells compared to T cells in air-exposed control rats [30]. Similarly, this group found the ratio of Th17/T_reg_ was significantly increased in smoke-exposed animals compared to air-exposed controls [30]. Hence, this Th17/T_reg_ axis is of increasing importance and may be common to many inhalation-induced lung diseases.

The lung microenvironment and its associated cytokine milieu is imperative in the differentiation and proliferation of certain inflammatory T cell phenotypes. There is evidence that pro-inflammatory signals within and surrounding the airways following epithelial injury impairs repair and promotes airway fibrosis. IL-17a acts directly on the airway epithelium as well as adjacent fibroblasts to induce pro-fibrotic cytokines [31]. Skewing of this local cytokine network has been implicated in the phenotypic plasticity of CD4^+^CD25^+^ T cells [32,33]. Particularly, cytokines in the IL-1 family are implicated in the differentiation of lung T cells toward a Th17 phenotype [34]. Future studies are underway by our lab to investigate the role of the lung microenvironment and its secreted cytokines in shifting this Th17/T_reg_ axis and how changing this proportion contributes to flavoring-related lung disease progression. More specifically, blocking either IL-1β or IL-36γ after DA inhalation exposure is of particular interest in attenuating lung disease progression.

Our study is not without a few limitations. First, significant differences exist in the anatomic structure of mice and rats. Specifically, the mouse lung lacks similar morphologic complexity and structure in comparison to rats for modeling human inhalation toxicology. Hence, the rat was used for modeling of flavoring-related lung disease but limited the number of reagents and antibodies available for T cell characterization. We failed to isolate Th17 cells directly, and thus used BALF IL-17a expression as an indirect assessment of Th17 cells within the lung. Second, a single concentration of diacetyl (200 ppm) was used in these studies. Extensive prior work has evaluated the dose response within the lung following DA inhalation exposure [9,11,12,13,35]. In our model, 200 ppm was previously determined to recapitulate human BO pathology in the Sprague-Dawley rats similar to prior rat DA exposure models [10,14]. Previous dosimetry modeling found this concentration (200 ppm DA) to be comparable to approximately 12 ppm exposure in humans [36], which is similar in range found in an occupational setting [37,38,39]. When lower concentrations are used in small rodents, significant scrubbing occurs within the nasal passages due to their high surface area, which ultimately prevents the majority of the DA to reach the intrapulmonary airways [13,36,40]. Third, the specific downstream effects and local cytokines that suppress the persistence of T_reg_ in the lungs were not identified but are currently under further investigation. Finally, there are risks associated with targeting this CD4^+^CD25^+^ T cell population as a potential therapy. Th17 cells are responsible for mitigating fungal infections and promoting barrier integrity within the lung, Hence, inhibiting Th17 cells may increase the risk of fungal infection or loss of mucosal integrity. Second, CD4^+^CD25^+^ T cells are known to express TGF-β. Overexpression of TGF-β may increase lung fibrosis through sustained or unbalanced proportions of Foxp3^+^ T cells in the lung.

In conclusion, repetitive exposure to DA vapors at occupationally relevant concentrations results in temporal skewing of the IL-17a/Foxp3 axis in the rat lung. Increased percent lung CD4^+^CD25^+^ T cells were present in DA-exposed rat lungs at 1 and 2 weeks post-exposure compared to air controls. Percent lung Foxp3^+^ T cells increased at 1 week post-exposure but did not persistent while BALF IL17a increased at 2 weeks, not seen at week 1. Increased CD4^+^CD25^+^ T cells and BALF IL-17a correlated with lower oxygen saturations and increased lung permeability, indicative of a potentially pathologic role of certain CD4^+^CD25^+^ T cells in the progression of flavorings-related lung disease.

## Figures and Tables

**Table 1:** Bronchi affected by extracellular matrix deposition and adjacent lymphoid hyperplasia after DA exposure.

Group (n = 5 Rats/ Time Point)	Extracellular Matrix Deposition /Rat Lung Section	Bronchial Lymphoid Aggregates /Rat Lung Section
Air Control	0.0 +/− 0.0	0.0 +/− 0.0
DA D5	0.8 +/− 0.5	1.6 +/− 0.5
DA D12	2.0 +/− 1.0	2.6 +/− 0.5
DA D19	2.6 +/− 1.5	3.0 +/− 0.0

## Data Availability

Not applicable.

## Supplementary Materials

The following are available online at https://www.mdpi.com/article/10.3390/toxics9120359/s1, Figure S1: Representative rat lung sections stained for Masson’s Trichrome demonstrating collagen I deposition (*blue*). (a) Room air (Control)-exposed rat airways, (b) DA Day 5 (immediately post-exposure) rat airways (c) DA Day 12 (1 week following DA exposures) rat airways, and (d) Day 19 (2 weeks post-exposure) rat airways. Organization of collagen I is noted in DA-exposed rats as early as Day 5 and persists to Day 19. Figure S2: Higher magnification image of hematoxylin & eosin (H&E) stained rat lung section at Day 12 post-diacetyl exposure. Mixed inflammatory infiltrate adjacent to DA affected airway with neutrophils (white arrow), eosinophils (black arrow), and clustered lymphocytes (arrow head) (bar: 25 µm). Figure S3: Rat lung sections stained for CD3, a T cell marker, and counterstained for hematoxylin in room air controls (a), DA-exposed Day 12 (b), and DA-exposed Day 19 (c) (bar: 250 µm). Inserts highlight the airway-centric, lymphoid aggregates with prominent CD3^+^ T cell staining within lymphoid aggregates (bar: 25 µm). Table S1: Flow Cytometry Antibody Panel.
