# Supplementary material for: Diacetyl Vapor Inhalation Induces Mixed, Granulocytic Lung Inflammation with Increased CD4+CD25+ T Cells in the Rat
Source: Toxics. 2021 Dec 20;9(12):359. doi: 10.3390/toxics9120359 (PMC8707442; doi:10.3390/toxics9120359)
Supplement: Supplementary file 1 [file toxics-09-00359-s001.zip › toxics-1482690-supplementary.pdf]

toxics

MDPI

Supplementary Materials: Diacetyl Vapor Inhalation Induces Mixed, Granulocytic Lung Inflammation with Increased CD4+CD25+ T Cells in the Rat

Emma L. House, So-Young Kim, Carl J. Johnston, Angela M. Groves, Eric Hernady, Ravi S. Misra, and Matthew D. McGraw

Table S1. Flow Cytometry Antibody Panel

Scheme 1. Flow Cytometry Markers.	Catalog #	Source	Fluorochrome	Dilution
**CD3**	201414	Biolegend	APC	1:150
**CD8**	25-0084-82	Invitrogen	PE-Cyanine 7	1:150
**CD4**	201518	Biolegend	APC-Cyanine 7	1:100
**CD25**	202103	Biolegend	Fitc	1:100
**FoxP3**	IC8970P	R&D	PE	1:100
**UltraComp Beads**	01-2222-42	Invitrogen		

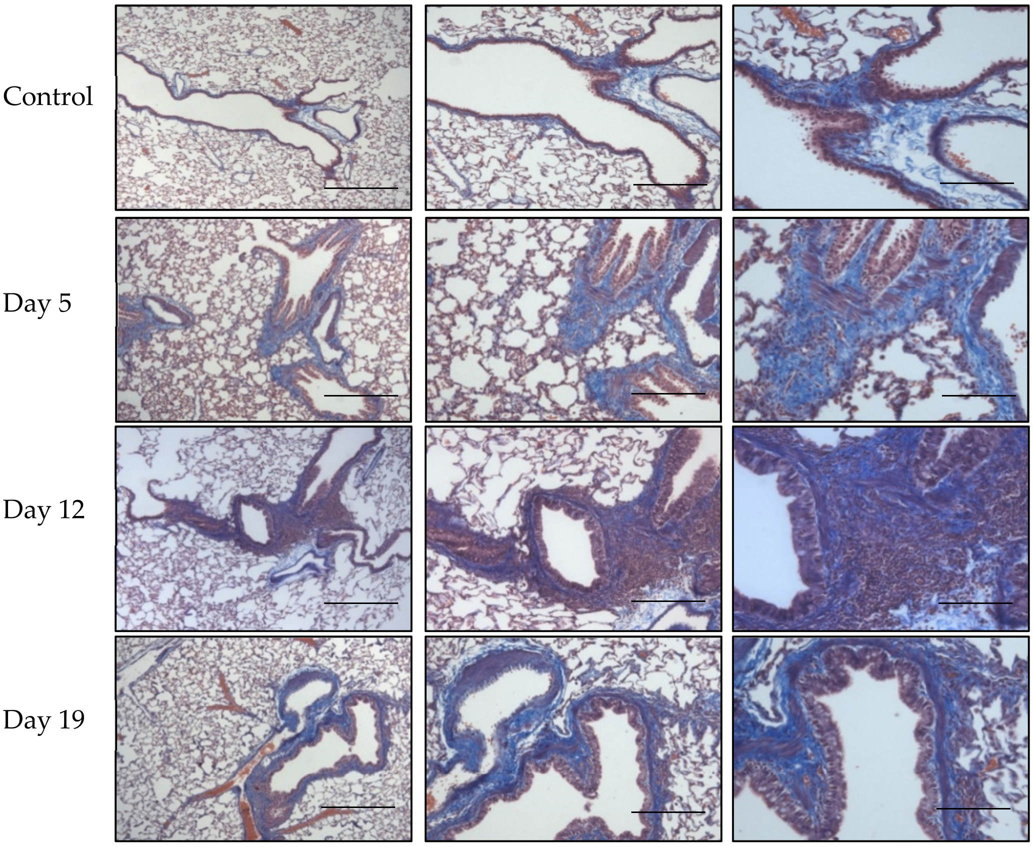

Figure S1. Representative rat lung sections stained for Masson's Trichrome demonstrating collagen I deposition (blue) (bar: 250 μm (left column), 75 μm (middle column), and 50 μm (right column)). (**a**) Room air (Control)-exposed rat airways, (**b**) DA Day 5 (immediately post-exposure) rat airways (**c**) DA Day 12 (1 week following DA exposures) rat airways, and (**d**) Day 19 (2 weeks post-exposure) rat airways. Organization of collagen I is noted in DA-exposed rats as early as Day 5 and persists to Day 19.

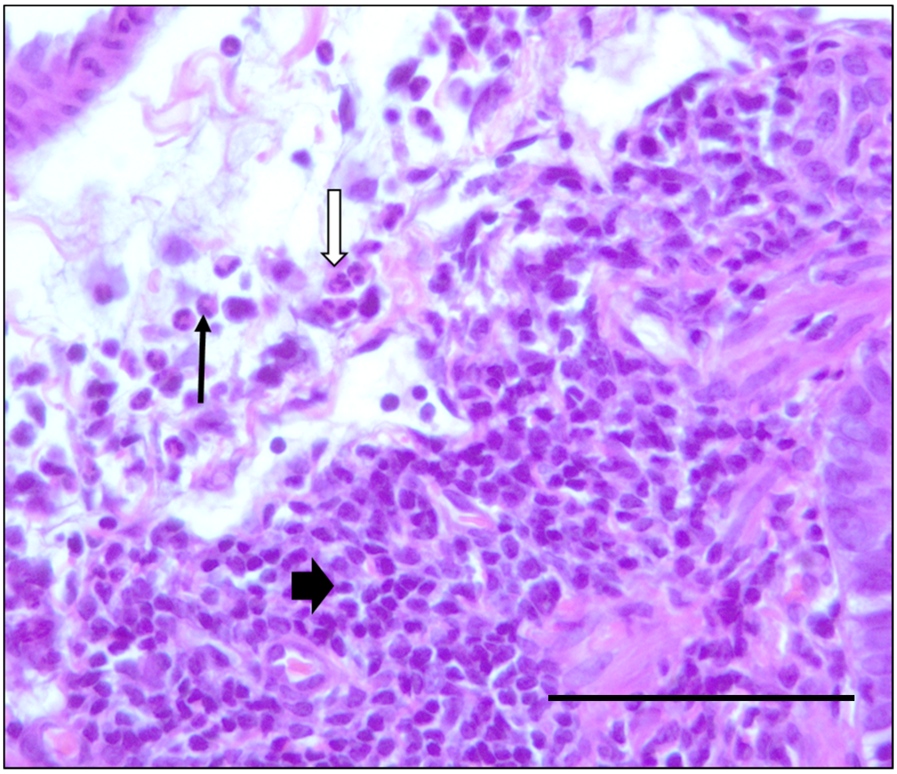

Figure S2. Higher magnification image of hematoxylin & eosin (H&E) stained rat lung section at Day 12 post-diacetyl exposure. Mixed inflammatory infiltrate adjacent to DA affected airway with neutrophils (white arrow), eosinophils (black arrow), and clustered lymphocytes (arrow head) (bar: 25 µm).

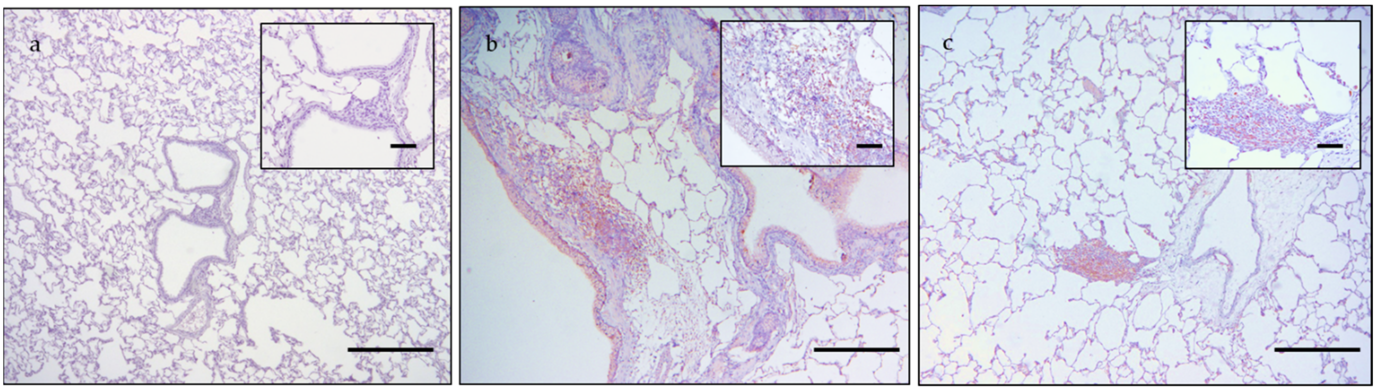

Figure S3. Rat lung sections stained for CD3, a T cell marker, and counterstained for hematoxylin in room air controls (**a**), DA-exposed Day 12 (**b**), and DA-exposed Day 19 (**c**) (bar: 250 µm). Inserts highlight the airway-centric, lymphoid aggregates with prominent CD3+ T cell staining within lymphoid aggregates (bar: 25 µm).
